# Supplementary material for: A cross-sectional study of functional and metabolic changes during aging through the lifespan in male mice
Source: eLife. 2021 Apr 20;10:e62952. doi: 10.7554/eLife.62952 (PMC8099423; doi:10.7554/eLife.62952)
Supplement: Figure 2—source data 1. [file elife-62952-fig2-data1.docx]

**Figure 2—Source data 1.** Multiple regression analysis results for Frailty Index and Gait Velocity.

| **Frailty Index** | | |
| --- | --- | --- |
|  | Models 1, 2, & 4 | Model 3 |
| Explanatory Variable | Parameter Estimate (p-value) | Parameter Estimate (p-value) |
| Intercept | 0.304499 (8.5E-05) | 0.343647 (0.0012) |
| Age | -0.0011 (0.6397) | -0.00805 (0.0157) |
| Gait Velocity | -0.72785 (0.0021) | -0.29512 (0.0424) |
| Glucose | -0.0004 (0.0218) | -0.00041 (0.0194) |
| HOMA-IR | 0.011247 (0.0012) | 0.014456 (5.1E-05) |
| Age× Gait Velocity | 0.03771 (0.0004) |  |
| Cortical Thickness |  | -0.75865 (0.0076) |
| Age× Cortical Thickness |  | 0.079253 (4.5E-06) |
| Model R^2^ | 0.766 | 0.829 |
|  |  |  |
| **Gait Velocity** | | |
|  | Models 1, 2, & 3 | Model 4 |
| Explanatory Variable | Parameter Estimate (p-value) | Parameter Estimate (p-value) |
| Intercept | 0.27946 (< 2E-16) | 0.0748 (0.3521) |
| Age | -0.00473 (2.7E-16) | 0.00241 (0.4614) |
| Energetic Costs |  | 0.000426 (0.0123) |
| Age× Energetic Costs |  | -1.55E-05 (0.0124) |
| Model R^2^ | 0.613 | 0.609 |

Note:

For Frailty Index, after the backward elimination, models 2 and 4 yielded the same final model as model 1. That is, the additional variables included for models 2 and 4 were not significant and were eliminated from the model.

For Gait Velocity, while Model 4 had more terms, the R^2^ is lower due to the sample sizes being different because of the smaller sample size of Energetic Cost data.
