# Supplementary material for: A cross-sectional study of functional and metabolic changes during aging through the lifespan in male mice
Source: eLife. 2021 Apr 20;10:e62952. doi: 10.7554/eLife.62952 (PMC8099423; doi:10.7554/eLife.62952)
Supplement: Figure 2—source data 2. [file elife-62952-fig2-data2.docx]

**Figure 2—Source data 2.** Area under the curves (AUCs) from the averaged, hourly trajectories of the indicated energetic parameters that were captured during two dark/light cycles in young (n=8), adult (n=22), and old (n=18) mice.

| Parameter | Age group | AUC | SEM | 95% CI |
| --- | --- | --- | --- | --- |
| VO_2_ (mL/hr) | Young  Adult  Old | 564.6  862.6**  574.1^†††^ | 51.35  51.61  44.18 | 464-665  761.4-963.7  487.5-660.7 |
| VCO_2_ (mL/hr) | Young  Adult  Old | 533.2  750.1*  639.2 | 44.29  43.3  48.55 | 446.4-620  665.2-834.9  544.1-734.4 |
| EE (kcal/hr) | Young  Adult  Old | 3.077  4.506**  3.255^††^ | 0.255  0.246  0.237 | 2.577-3.576  4.024-4.989  2.789-3.72 |
| Ambulation (counts) | Young  Adult  Old | 11072  9827  5802^§,†^ | 1137  1228  771.4 | 8843-13301  7419-12234  4290-7314 |
|  |  |  |  |  |

*, **, *** p< 0.05, < 0.01, < 0.001 (Adult vs. Young)

^†^, ^††^, ^†††^ p< 0.05, < 0.01, < 0.001 (Old vs. Adult)

^§^, p< 0.05 (Old vs. Young).
