# Supplementary material for: A cross-sectional study of functional and metabolic changes during aging through the lifespan in male mice
Source: eLife. 2021 Apr 20;10:e62952. doi: 10.7554/eLife.62952 (PMC8099423; doi:10.7554/eLife.62952)
Supplement: Figure 2—source data 3. [file elife-62952-fig2-data3.docx]

**Figure 2—Source data 3.** Distribution of various energetic parameters in male mice in various age groups.

| Parameter | Age group | mean | SEM | 95% CI |
| --- | --- | --- | --- | --- |
| VO_2_ (mL/hr) | Young  Adult  Old | 119.1  132.7*  119.9^††^ | 4.265  2.19  2.442 | 109-129.2  128.1-137.2  114.7-125 |
| VCO_2_ (mL/hr) | Young  Adult  Old | 102.8  112.9  107.6 | 3.53  1.716  2.58 | 94.5-111.2  109.4-116.5  102.1-113 |
| EE (kcal/hr) | Young  Adult  Old | 0.581  0.647*  0.591^††^ | 0.0203  0.0096  0.0127 | 0.5328-0.629  0.627-0.667  0.5637-0.6174 |
| Ambulation (counts) | Young  Adult  Old | 450.8  420.7  249.9^§,††^ | 63.56  42.19  31.77 | 300.5-601.1  332.4-508.4  182.9-316.9 |
|  |  |  |  |  |

*, **, *** p< 0.05, < 0.01, < 0.001 (Adult vs. Young)

^†^, ^††^, ^†††^ p< 0.05, < 0.01, < 0.001 (Old vs. Adult)

^§^, p< 0.05 (Old vs. Young).
