# Supplementary material for: A cross-sectional study of functional and metabolic changes during aging through the lifespan in male mice
Source: eLife. 2021 Apr 20;10:e62952. doi: 10.7554/eLife.62952 (PMC8099423; doi:10.7554/eLife.62952)
Supplement: Figure 2—source data 4. [file elife-62952-fig2-data4.docx]

**Figure 2—Source data 4.** Linear regression of the metabolic cage data obtained from the whole cohort of male mice and after segregation by age groups.

| Correlation (n=48) | r^2 | p |  |  |  |  |
| --- | --- | --- | --- | --- | --- | --- |
| VO_2_ vs. BW | 0.4516 | <0.0001*** |  |  |  |  |
| VO_2_ vs. Age | 0.0205 | 0.3316 |  |  |  |  |
| VCO_2_ vs. BW | 0.09373 | 0.0343* |  |  |  |  |
| VCO_2_ vs. Age | 0.00076 | 0.8525 |  |  |  |  |
| EE vs. BW | 0.3689 | <0.0001*** |  |  |  |  |
| EE vs. Age | 0.0114 | 0.4691 |  |  |  |  |
| Ambulation vs. BW | 0.1001 | 0.0284* |  |  |  |  |
| Ambulation vs. Age | 0.3149 | <0.0001*** |  |  |  |  |
|  |  |  |  |  |  |  |
|  | Young (n=8) | | Adult (n=22) | | Old (n=18) | |
| Correlation | r^2 | p | r^2 | p | r^2 | P |
| VO_2_ vs. BW | 0.898 | 0.0003*** | 0.3711 | 0.0026** | 0.03268 | 0.4729 |
| VCO_2_ vs. BW | 0.8498 | 0.0011** | 0.01173 | 0.6314 | 0.0033 | 0.8209 |
| EE vs. BW | 0.9179 | 0.0002*** | 0.0387 | 0.0090** | 0.0093 | 0.7028 |
| Ambulation vs. BW | 0.4292 | 0.0778 | 0.0551 | 0.2932 | 0.1155 | 0.1677 |
|  |  |  |  |  |  |  |

Significance: * <0.05; ** <0.01; ***; <0.001.
