# Supplementary material for: A cross-sectional study of functional and metabolic changes during aging through the lifespan in male mice
Source: eLife. 2021 Apr 20;10:e62952. doi: 10.7554/eLife.62952 (PMC8099423; doi:10.7554/eLife.62952)
Supplement: Figure 2—source data 5. [file elife-62952-fig2-data5.docx]

**Figure 2—Source data 5**. Generalized linear model (GLM) to assess potential significant interaction effect in energetic parameters between mice of various age groups.

|  | GLM (Adult vs. Young) | | | GLM (Old vs. Young) | | |
| --- | --- | --- | --- | --- | --- | --- |
| Effect | Mass | Group | Interaction | Mass | Group | Interaction |
| VO_2_ (mL/hr) | <0.001*** | 0.0271* | 0.0404* | <0.001*** | 0.0188* | 0.0242* |
| VCO_2_ (mL/hr) | 0.0080** | 0.0118* | 0.0202* | 0.0080** | 0.0137* | 0.0211* |
| EE (kcal/hr) | 0.0014** | 0.0200* | 0.0320* | 0.0014** | 0.0142* | 0.0194* |
|  |  |  |  |  |  |  |
|  | Adult vs. Young | | | Old vs. Young | | |
| RER | 0.5877 | | | 0.1109 | | |
| Locomotor activity (beam breaks) | 0.6855 | | | 0.1165 | | |
| Ambulatory activity (beam breaks) | 0.5670 | | | 0.0064** | | |

Data were generated from the CalR software (Mina et al., 2018). Significance: <0.001, ***; <0.01, **; <0.05, *.
